# Is retrograde menstruation a universal, recurrent, physiological phenomenon? A systematic review of the evidence in humans and non-human primates

**DOI:** 10.1093/hropen/hoae045

**Published:** 2024-07-12

**Authors:** Paola Viganò, Francesca Caprara, Francesca Giola, Giorgia Di Stefano, Edgardo Somigliana, Paolo Vercellini

**Affiliations:** Infertility Unit, Fondazione IRCCS Ca’ Granda Ospedale Maggiore Policlinico, Milan, Italy; Department of Clinical Sciences and Community Health, Academic Center for Research on Adenomyosis and Endometriosis, Università degli Studi di Milano, Milan, Italy; Department of Clinical Sciences and Community Health, Academic Center for Research on Adenomyosis and Endometriosis, Università degli Studi di Milano, Milan, Italy; Infertility Unit, Fondazione IRCCS Ca’ Granda Ospedale Maggiore Policlinico, Milan, Italy; Infertility Unit, Fondazione IRCCS Ca’ Granda Ospedale Maggiore Policlinico, Milan, Italy; Department of Clinical Sciences and Community Health, Academic Center for Research on Adenomyosis and Endometriosis, Università degli Studi di Milano, Milan, Italy; Infertility Unit, Fondazione IRCCS Ca’ Granda Ospedale Maggiore Policlinico, Milan, Italy; Department of Clinical Sciences and Community Health, Academic Center for Research on Adenomyosis and Endometriosis, Università degli Studi di Milano, Milan, Italy

**Keywords:** endometriosis, menstruation, ovulation, endometrium, blood, iron, uterus

## Abstract

**STUDY QUESTION:**

What are the quantitative, qualitative, and temporal patterns of retrograde mentruation?

**SUMMARY ANSWER:**

The extreme quantitative and qualitative heterogeneity of the available studies prevents the definitive conclusion that retrograde menstruation is a universal and consistent phenomenon during the reproductive period.

**WHAT IS KNOWN ALREADY:**

Retrograde menstruation has been defined as a universal, physiological phenomenon that occurs similarly in about 90% of menstruators during the reproductive period. However, uncertainties still exist in terms of the event frequency, total amount, and cellular composition of retrograde menstruation and the differences between individuals with versus those without endometriosis.

**STUDY DESIGN, SIZE, DURATION:**

Two systematic reviews were performed, one for human studies, and one for non-human primate studies. We retrieved studies from the PubMed and Embase databases published between 1 January 1980 and 1 November 2023. Studies published in the English language were included and identified using a combination of MeSH terms. References from relevant publications were systematically screened and further articles were identified using PubMed’s ‘similar articles’ and ‘cited by’ functions.

**PARTICIPANTS/MATERIALS, SETTING, METHODS:**

Results were reported in accordance with the PRISMA guidelines. Studies that did not report original data or provided a review of the field were excluded. Bias analysis was completed for each included human study by using the Newcastle–Ottawa scoring system.

**MAIN RESULTS AND THE ROLE OF CHANCE:**

Fifteen studies were finally included in the human systematic review, mostly with limited sample sizes. The macroscopic visualization of blood in PF during menses was reported with a frequency ranging from 9% to 100%. A prevalence of endometrial cells detected in peritoneal fluid ranging from 8% to 75% was reported in the various studies. Controversial findings were reported in relation to patients with endometriosis. Retrograde menstruation has been evaluated cross-sectionally on single occasions, and no information is available on the course of the phenomenon within an entire cycle and between subsequent cycles. Two studies were included in the non-human primate systematic review; one of them showed that retrograde menstruation was observed more frequently in baboons with naturally occurring endometriosis (83%) than in those with a normal pelvis (51%).

**LIMITATIONS, REASONS FOR CAUTION:**

In humans, peritoneal fluid has often been collected at different cycle phases and not systematically during menstruation. The indication for laparoscopy was not always clear for all participants. A wide variety of methods were used to detect endometrial cells, including cytological staining, cell block analysis, immunocytochemistry, and various methods of cell culture.

**WIDER IMPLICATION OF THE FINDINGS:**

The idea that almost all women experience retrograde menstruation regularly and similarly during their reproductive life is currently unsubstantiated. It is an academic notion accepted uncritically. Development of endometriosis may derive from differences in the frequency or severity of the event.

**STUDY FUNDING/COMPETING INTEREST(S):**

The review was partially funded by Italian Ministry of Health—Current Research IRCCS. P.Vi. serves as co-editor in Chief of Journal of Endometriosis and Uterine Disorders. E.S. serves as Editor in Chief of Human Reproduction Open and discloses research grants from Ferring, Ibsa, Gedeon Richter, and Theramex, and honoraria from Ibsa and Gedeon Richter. P.Ve. serves as Associate Editor for Human Reproduction Open; is a member of the Editorial Board of the Journal of Obstetrics and Gynaecology Canada, of the Italian Journal of Obstetrics and Gynaecology, and of the International Editorial Board of Acta Obstetricia et Gynecologica Scandinavica; has received royalties from Wolters Kluwer for chapters on endometriosis management in the clinical decision support resource UpToDate; and maintains both a public and private gynecological practice. All other authors declare they have no conflict of interest.

**REGISTRATION NUMBER:**

N/A.

WHAT DOES THIS MEAN FOR PATIENTS? Retrograde menstruation, which occurs when menstrual blood and uterine tissue reach the peritoneal cavity through the fallopian tubes, has been defined as a universal phenomenon happening consistently in about 90% of female subjects during the reproductive period. This phenomenon is considered critical in the sequence of events leading to endometriosis. Although interest in retrograde menstruation dates back almost a century, uncertainties remain regarding the frequency of this event during the menstrual phase in the general population and the amount of blood and tissue refluxed. More importantly, further evidence is needed regarding potential differences in the incidence, quantity, and quality of retrograde menstruation between individuals with and without endometriosis. Gathering current research on the prevalence of retrograde menstruation in endometriosis in both humans and non-human primates, we have found that the results from available studies are highly heterogeneous. Blood was observed in the pelvis during menses with a frequency ranging from 9% to 100% according to various studies. Controversial findings were also reported concerning the quantity of tissue present and the frequency in women with endometriosis. The claim that almost all women experience regular and similar retrograde menstruation during the reproductive period may be hypothetically true, but it is currently not supported by the available data. Therefore, one of the most widely accepted models to explain the development of endometriosis should be revisited.

## Introduction

In 1986, Liu and Hitchcock wrote: ‘Many theories have been advanced both for the presence of endometriosis and for its associated complications […]. The contribution of retrograde menstruation is still uncertain’ (Liu and Hitchcock, 1986). Recently, Allaire *et al.* stated: ‘Many theories have been suggested to explain the development of endometriosis, but none are definitive. The most accepted theory is that endometrial cells reach the peritoneal cavity through retrograde menstruation’ (Allaire *et al.*, 2023). At first glance, the uninitiated reader might conclude that these two statements were written at about the same time. But almost 40 years have passed since then. What has happened in between? Why is it that no definitive information seems to have been obtained about the role of retrograde menstruation (RM) in the pathogenesis of endometriosis? Indeed, RM has been defined as a ‘physiological process’ and its ‘physiological mechanisms’ have been described in detail by highly authoritative researchers (Filby *et al.*, 2020). But is the popular claim that RM occurs similarly in 90% of women (Filby *et al.*, 2020; Allaire *et al.*, 2023) a proven fact?

Indeed, the RM phenomenon would need to be described quantitatively and qualitatively. Can a physiological level of transtubal reflux be defined? Are endometrial cells or fragments systematically present in tubal spillage? What are the proportions of the cellular components of RM? Is the amount of erythrocytes and endometrial cells fairly stable and similar over time in most menstruators? Do data exist demonstrating that RM is a regularly repetitive phenomenon during different reproductive periods? Finally, are the above variables comparable in women with and without endometriosis? Only in the latter case could retrograde menstruation be defined as a physiological process; otherwise, this phenomenon by itself would assume the characteristics of a causative pathogenic factor, and would challenge the common tenet that other causative factors in addition to RM are absolutely necessary to explain the onset and progression of endometriosis. In the words of Bokor *et al.* (2009), ‘the lack of knowledge regarding potential differences in the presence and distribution of PF [peritoneal fluid] cell populations during menstruation between women with and without endometriosis is a major obstacle with respect to the validity of the Sampson hypothesis’ (Bokor *et al.*, 2009).

Interest in RM dates back almost a century to the original debate between Sampson and Novak regarding the pathogenic role of transtubally refluxed endometrial cells (Novak, 1926, 1936; Sampson, 1927, 1940). However, it was not until the 1980s that the topic regained worldwide interest based on the results of formal studies that went beyond anecdotal findings and expert opinion. Therefore, with the aim of trying to partially disentangle the above uncertainties, we deemed it useful to critically evaluate the literature data on RM published since 1980.

The main objective of this systematic review was to try to understand whether it is currently possible to define the phenomenon of RM in terms of: (i) the frequency of the event during the menstrual phase of the cycle in the general population; (ii) the total amount of transtubally refluxed menstruation (quantitative assessment); (iii) the cellular composition of transtubally refluxed menstruation and prevalence of endometrial cells, glands, or tissue fragments (qualitative assessment); and (iv) the differences in incidence, quantity, and quality of RM between individuals with and without endometriosis.

## Methods

These two systematic reviews have been conducted according to the Preferred Reporting Items for Systematic Review and MetaAnalyses (PRISMA) guidelines.

### Systematic search

We searched the PubMed or Embase databases between 1 January 1980 and 1 November 2023. According to the perspective of Filby *et al.* (2020), we focused here on the *product* rather than the *process* of RM. Therefore, potential anatomical or functional determinants of RM were not specifically addressed. The search string for the search in humans was the following: (endometriosis) AND ((retrograde menstruation) OR (tubal reflux) or (peritoneal fluid) OR (pelvic fluid) OR (pelvic effusion)) and the filtering for ‘human’ was applied. The same search string was used for the search in animals and the filtering for ‘animal’ was applied.

### Inclusion and exclusion of studies

The search was limited to full-length articles published in English-language peer-reviewed journals between 1 January 1980 and 1 November 2023. Articles in humans were excluded if: (i) the aim was not related to menstruation (e.g. Kulenthran and Jeyalakshmi, 1989; van der Weiden RM *et al.*, 1992); (ii) the nature of menstrual effluent samples could not be clarified based on the molecular analysis performed (e.g. van der Linden *et al.*, 1994), or (iii) based on the data provided it was not possible to deduce the rate of RM in women with and without the disease (e.g. Tang *et al.*, 2022). Studies in animals were excluded if they did not report any information about RM (Davis *et al.*, 1973; D’Hooghe *et al.*, 2001) or if RM was induced and not spontaneous (D’Hooghe *et al.*, 1994, 1995). Studies that did not report original data or studies that provided a review of the field only were excluded. Abstracts presented at meetings were not considered.

### Study selection

Results from the initial searches were collated, and duplicates were deleted. Literature searches were performed by three researchers (F.C., F.G., and G.D.S.). Differences of opinion in the team were solved by discussion and consensus. References from relevant publications were systematically screened and further articles were identified using PubMed’s ‘similar articles’ and ‘cited by’ functions.

### Data extraction and synthesis

The extracted data included but were not limited to title, author, journal, year of publication, population studied, interventions, results, comparisons, and outcomes. Given the heterogeneity of the methods and results found throughout this review, no statistical meta-analysis was possible.

### Appraisal of quality of evidence

The Newcastle–Ottawa scale (NOS) was used to assess the quality of human studies included in this review.

## Results

### Frequency, amount, and composition of RM and differences between women with and without endometriosis

**Figure 1.**
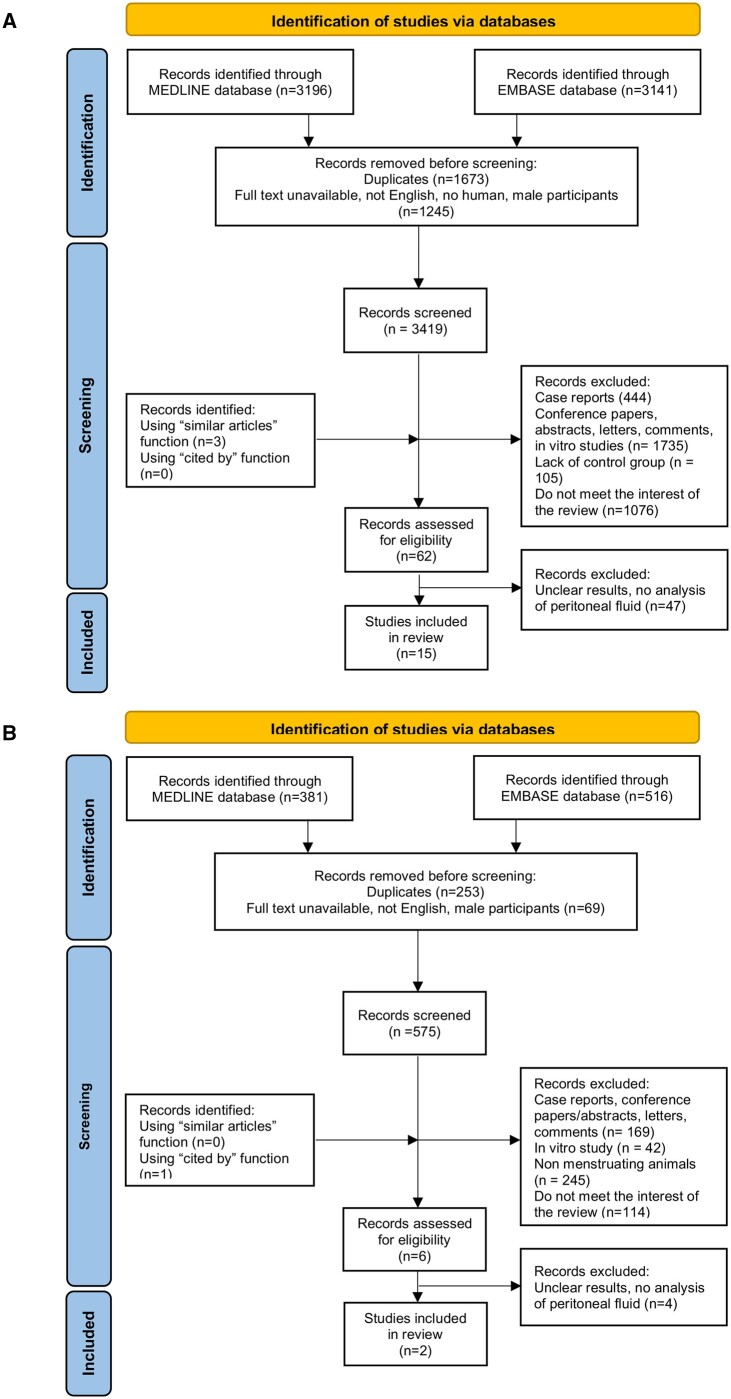
PRISMA flow diagram of the literature search and selection process for humans (A) and animal studies (B).

A total of 15 original studies in humans were finally selected for this review: eight published in the 1980s, two in the 1990s, three in the 2000s, one in 2017, and one in 2021. The identification and selection process in humans are shown in Fig. 1A. Five studies were conducted in the USA, three in Belgium, three in the Netherlands, two in Australia, one in Italy, and one in the UK (Table 1). Outcomes measured, relevant findings, and conclusions drawn are summarized in Table 2.

**Table 1.** Summary of characteristics and designs of the included human studies.

Author, year	Country	Human/animal	Method	Cases with endometriosis (n)/controls (n)
Koninckx *et al.* (1980)	Belgium	Human	Papanicolaou staining of cells in PF	37/44
Blumenkrantz *et al.* (1981)	USA	Human	Visual inspection of blood contaminated PF	11 women undergoing intraperitoneal dialysis; no cases with endometriosis.
Reti *et al.* (1983)	Australia	Human	Visual inspection of blood and Papanicolaou staining of cells in PF	15 patients with severe dysmenorrhea or abdominopelvic pain/31 asymptomatic patients undergoing laparoscopic tubal diathermy
Halme *et al.* (1984)	USA	Human	Visual categorization of the PF as ‘straw’, ‘pink’, or ‘bloody’	81/221
Badawy *et al.* (1984)	USA	Human	Papanicolaou staining of cells in PF	45/57
Willemsen *et al.* (1985)	The Netherlands	Human	PF cell culture before and after tubal irrigation	4/111
Liu and Hitchcock (1986)	UK	Human	Laparoscopic visual observation of PF	32/43
Bartosik *et al.* (1986)	USA	Human	Papanicolaou staining and block preparation of cells in PF before and after uterine irrigation	52/15
Kruitwagen (1991)	The Netherlands	Human	PF cell culture before and after tubal irrigation cell culture	12/12
van der Linden *et al.* (1995)	The Netherlands	Human	Immunohistochemical analysis of cells in PF	8/8
Bulletti *et al.* (2002)	Italy	Human	PF cell culture	22/22
Sharpe-Timms (2005)	USA	Human	PF cell culture, histological confirmation of endometrial glands/stroma, evaluation of haptoglobin gene expression, and protein localization	65/34
Bokor *et al.* (2009)	Belgium	Human	Papanicolau staining of cells in PF and immunocytochemical analysis	19/13
Dorien *et al.* (2017)	Belgium	Human	Papanicolau staining of cells in PF and immunocytochemical analysis	9/8
Masuda *et al.* (2021)	Australia	Human	Clonogenicity assay and flow cytometry of cells in PF	32/29

PF, peritoneal fluid.

**Table 2.** Summary of the outcomes measured, relevant findings, and conclusions drawn from the included human studies.

Author, year	Outcome measure	Relevant findings	Conclusion
Koninckx *et al.* (1980)	EC in PF	EC present in 44% of women, both with and without endometriosis, most frequently during the follicular phase	EC in PF derived from retrograde menstruation and not from endometriotic lesions, since frequently present in both women with and without endometriosis
Blumenkrantz *et al.* (1981)	Blood and/or EC in PF in the peritoneal dialysis catheter.	Blood present in 81%. No EC cells in PF from three patients.	Retrograde menstrual bleeding into the peritoneal cavity is the rule rather than the exception.
Reti *et al.* (1983)	Blood and/or EC in in the pouch of Douglas.	Blood present in 67% symptomatic and in 74% asymptomatic women. EC present in 17% of women. One case of endometriosis without blood in Douglas.	Transtubal blood reflux is associated with perimenstrual pain, but not with endometriosis. The visualization of blood in the pouch of Douglas at laparoscopy does not confirm retrograde menstruation as this may have collected from the trauma of entry.
Halme *et al.* (1984)	Blood cells in PF	Non-menstrual phase: pink or bloody fluids in 61% with patent tubes and in 60% with occluded tubes. Perimenstrual phase: bloody fluids in 90% with patent tubes (90% women with endometriosis + 90% women without) and in 15% with occluded tubes.	Retrograde menstruation through the fallopian tubes is common occurrence in all menstruating women with patent tubes. No significant difference in the frequency of blood in PF on perimenstrual days between cases and controls.
Badawy *et al.* (1984)	EC in PF	EC present in 31% of women with and in 8% without endometriosis	Changes in the peritoneal environment in endometriosis may lead to infertility
Willemsen *et al.* (1985)	Epithelial cells in PF before and after tubal irrigation.	No epithelial cells in peritoneal washings before flushing, even in patients with endometriosis. Epithelial cells in 67% of cultures after uterine/tubal flushes.	The presence of epithelial cells in the peritoneal cavity after flushes may supports Sampson’s theory of endometriosis.
Liu and Hitchcock (1986)	Heavy coating of the pelvic organs with dark menstrual blood to blood staining of the PF in the pouch of Douglas	Retrograde menstruation in 76% of patients during the menstrual phase. Retrograde menstruation in 97% of endometriosis patients. Of the patients with retrograde menstruation, 54% had endometriosis (Stage I).	Association between retrograde menstruation and endometriosis.
Bartosik *et al.* (1986)	EC in PF	Before uterine irrigation, EC present in 19% of patients with endometriosis and in 11% without the disease. After uterine irrigation, 76% versus 42%, respectively.	No significant difference in presence of endometrial tissue between patients with and without endometriosis. Higher concentration of EC only after uterine irrigation in women with endometriosis than controls.
Kruitwagen *et al.* (1991)	EC colonies from culture of epithelial cells in PF	EC colonies present in 79% of women, 67% with endometriosis and 92% without.	Retrograde transport of viable EC during menstruation occurs in most women with patent fallopian tubes without significant difference in the incidence and number of cell colonies between women with and without endometriosis.
van der Linden *et al.* (1995)	Immunohistochemical characteristics of epithelial cells in PF	No significant differences in immunohistochemical characteristics in women with and without endometriosis.	Transport of menstrual debris into peritoneal cavity in women with patent tubes is supported.
Bulletti *et al.* (2002)	Blood and viable EC in PF	Retrograde bleeding in 73% of patients with endometriosis and in 9% of controls. EC present in 45% of patients with endometriosis.	Endometriosis is significantly associated with both retrograde bleeding and presence of viable epithelial/stromal EC in the cul-de-sac.
Sharpe-Timms (2005)	Histologically confirmed endometrial glandular epithelial and stromal cells.	Proportion of peritoneal fluids containing tissue fragments greater in women with endometriosis (43%) than in women without endometriosis (15%). When tissue fragments were present in PF, no statistically significant difference in endometrial glands in women with (46%) and without (60%) endometriosis.	More visible tissue fragments are isolated from PF of women with endometriosis than from PF of women without endometriosis.
Bokor *et al.* (2009)	Red blood cells, white blood cells and EC detected by immunocytological staining in PF.	In menstrual phase, higher concentration of leucocytes and erythrocytes than in non-menstrual phase of the cycle. Low concentration of EC in all phases of the cycle (12.5% of the subjects). No difference in immunocytological profile between cases and controls.	The prevalence and/or amount of EC in the PF is not increased in women with endometriosis or during menstruation. Menstruation is associated with a higher number of leukocytes and erythrocytes.
Dorien *et al.* (2017)	Epithelial EC, Stromal EC, mesothelial cells detected by immunocytological staining in PF during the menstrual phase.	Epithelial EC present in 56% of cases and in 75% of controls. Stromal EC present in 67% of cases and in 38% controls.	No significant difference in the prevalence of epithelial and stromal EC between cases and controls.
Masuda *et al.* (2021)	Clonogenic EC, SUSD2+ mesenchymal stem cells and N-cadherin+ epithelial progenitor cells in menstrual blood, PF and peripheral blood.	During menstruation, endometrial mesenchymal stem cells present in 77% of cases and 44% of controls, epithelial progenitor cells in 60% of cases and in 25% of controls. More clonogenic cells present beyond the menstrual phase in women with endometriosis than in the control group.	The study supports both the Sampson’s and stem cell hypotheses.

PF, peritoneal fluid; EC, endometrial cells.

Five studies reported on the macroscopic visualization of blood in the peritoneal cavity during menstruation in humans (Blumenkrantz *et al.*, 1981; Reti *et al.*, 1983; Halme *et al.*, 1984; Liu and Hitchcock, 1986; Bulletti *et al.*, 2002). The presence of blood in peritoneal fluid (PF) during menses was reported with a frequency ranging from 9% to 100%. Controversial findings derived from the comparison between women with and without endometriosis. Reti *et al.* (1983) reported that the native PF of 46 women was bloody in 10 out of 15 (67%) women with severe dysmenorrhea or abdominopelvic pain and in 23 out of 31 (74%) asymptomatic women undergoing tubal diathermy. Halme *et al.* (1984) limited their investigation to the visual categorization of the native PF aspirated during laparoscopy as ‘straw’, ‘pink’, or ‘bloody’. When the PF was obtained during the perimenstrual period, its color was pink or bloody in 9 out of 10 participants with endometriosis, but also in 38 out of 42 (90%) participants without endometriosis. However, the PF was judged pink or bloody in only two of 13 (15%) patients with tubal obstruction. Thus, these findings support the very high frequency of retrograde blood flow and the role of tubal patency in this phenomenon but do not demonstrate a difference in the frequency of bloody PF between individuals with and those without endometriosis (Halme *et al.*, 1984). Different findings were observed by Liu and Hitchcock (1986) when they performed laparoscopic sterilization during menstruation in predominantly parous women. Retrograde menstruation, defined as heavy coating of the pelvic organs with dark menstrual blood or blood staining of the PF in the pouch of Douglas, was observed in 76% of participants. Early superficial peritoneal endometriosis was detected in 32 (43%) individuals, 31 of whom were in the RM group (31/57, 54% versus 1/18, 6%). Retrograde menstruation was not associated with dysmenorrhea or menorrhagia (Liu and Hitchcock, 1986).

Blood in the peritoneum was macroscopically detected in 73% of the individuals with endometriosis but only in 9% of those without the disease by Bulletti *et al.* (2002). The presence of blood in the peritoneal effluent based on visual inspection only was also the outcome measure of the study by Blumenkrantz *et al.* (1981). The authors reported the presence of blood in the silicone tubes used for intraperitoneal dialysis immediately before and during menstruation in 9 of 11 women younger than 45 years with severe renal failure. While all previous reports have described occasional findings at a single time point, this is the first study demonstrating the serial presence of blood in the abdominal cavity with each repeated menstruation. However, attempts to identify endometrial cells or glands in the peritoneal effluent of three patients were unsuccessful. Moreover, no intra-abdominal endometriotic lesions were observed in six patients who underwent laparotomy. Additionally, the study was retrospective and conducted on a very limited number of women who were mostly amenorrheic due to their medical condition prior to starting dialysis. Finally, because laparotomy was undertaken to remove the kidneys and/or spleen prior to kidney transplantation, the procedure was performed by surgeons with likely limited awareness or interest in pelvic endometriosis; thus, underreporting cannot be excluded (Blumenkrantz *et al.*, 1981).

A cytological analysis using Papanicolau staining of cells isolated from PF samples collected during laparoscopy was performed in six studies (Koninckx *et al.*, 1980; Reti *et al.*, 1983; Badawy *et al.*, 1984; Bartosik *et al.*, 1986; Bokor *et al.*, 2009; Dorien *et al.*, 2017). In these studies, endometrial cells have been found in the PF of menstruators with a frequency ranging from 8% to 75%. The highest frequency was found when the Papanicolau staining was associated with immunocytochemical assessments (Dorien *et al.*, 2017). A single study reported a strong difference in the frequency of PF endometrial cells between women with and without endometriosis (Badawy *et al.*, 1984). Koninckx *et al.* (1980) observed a 44% frequency of endometrial cells in PF mostly during the follicular phase without differences between affected and non-affected women. However, only four women with endometriosis underwent surgery in the follicular phase, and heavily blood-stained samples were discarded (Koninckx *et al.*, 1980). Reti *et al.* (1983) detected endometrial glandular structures in 17% of women undergoing laparoscopy during menses and in 24% of those with blood-stained PF. Importantly, as previously mentioned, the authors’ mere visualization of blood in PF resulted in completely different findings (Reti *et al.*, 1983). According to Badawy *et al.* (1984), endometrial cells, detected as conglomerate groups of columnar epithelial cells, were present in the PF of 14 (31%) individuals in the endometriosis group and in 5 (8%) of the non-endometriosis group (Badawy *et al.*, 1984). The cycle phase was, however, not reported. Bartosik *et al.* (1986) collected native PF from the Douglas pouch both before and after uterine irrigation. Patients with tubal obstruction were excluded. Before uterine irrigation, endometrial cells were identified in 6 of 32 (19%) patients with endometriosis and in 1 of 9 (11%) without the disease. The proportions after uterine irrigation were 76% versus 42%, respectively (Bartosik *et al.*, 1986).

Bokor *et al.* (2009) performed laparoscopy for pelvic pain and/or infertility in 50 patients with minimal to severe endometriosis and 48 subjects with a normal pelvis, and evaluated variations in native PF red blood cells, white blood cells, and endometrial cells throughout the menstrual cycle in the two study groups. In a subset of 32 participants (19 with and 13 without endometriosis), the different cell types were identified using Papanicolau staining and immunocytochemical analysis. Compared to the non-menstrual phase cycle, the analysis of PF collected on menstrual days showed a 13-, 8-, and 10-fold increase in erythrocytes, hemoglobin, and hematocrit, respectively. Cells with an endometrial phenotype were detected in only one out of eight individuals (4/32 = 12.5%), without differences between the different phases of the cycle (menstrual phase, 1/7; follicular phase, 1/5; luteal phase, 2/20). The authors could not confirm that the prevalence and amount of PF endometrial cells are increased in patients with endometriosis compared to controls or generally during menstruation compared to other phases of the cycle (Bokor *et al.*, 2009).

Dorien *et al.* (2017) retrospectively analyzed previously stored PF samples on patients who underwent laparoscopy during the menstrual phase for pain or infertility. Overall, in cases and controls, the prevalence of PF epithelial (56% versus 75%, respectively) and stromal (67% versus 38%, repectively) endometrial cells on Papanicolau and immunocytochemical staining did not differ (Dorien *et al.*, 2017).

A similar design using hematoxylin and eosin staining for histological analysis of peritoneal tissue fragments was used by Sharpe-Timms (2005) who collected PF during tubal sterilization. Histologic examination of cell blocks identified endometrial glands and stroma in 20% of patients with endometriosis and 9% of controls, with no significant differences between PF samples collected at different cycle phases (Sharpe-Timms, 2005).

Three studies used cell culture to identify endometrial cells in PF reporting controversial findings. Different findings were also derived from the comparison between women with and without endometriosis. Willemsen *et al.* (1985) collected PF before and after tubal irrigation in infertile subjects undergoing laparoscopy in the preovulatory phase. Uterine and tubal epithelial cells could be cultured from the peritoneal cavity obtained after flushing in 77 individuals, but in no case when the native PF was aspirated before flushing, even when endometriosis was present (Willemsen *et al.*, 1985). The Nijmegen group replicated the previous study published in 1985 (Willemsen *et al.*, 1985), but this time performed laparoscopy on the 24 infertile participants in the early follicular phase (cycle Days 1–7) instead of the preovulatory phase. After culturing cell pellets obtained from PF prior to tubal flushing, endometrial cell colonies were observed in 19 (79%) cases: 8/12 (67%) in patients with endometriosis and 11/12 (92%) in those without the disease. No significant difference in the number of cell colonies was observed between the two groups (Kruitwagen *et al.*, 1991). Bulletti *et al.* (2002) recovered and cultured monolayers of epithelial and stromal components from PF collected during menses. Endometrial cells were isolated from 45% of patients with endometriosis and from none of the control subjects (Bulletti *et al.*, 2002).

To verify the endometrial origin of epithelial cells retrieved from native PF on Days 2–5 of the cycle, van der Linden *et al.* (1995) evaluated their immunohistochemical characteristics with those of cells from the menstrual effluent and eutopic endometrium. Red blood cells and epithelial cells were identified in all PF samples. No significant differences were observed in the immunohistochemical characteristics of cells from menstrual effluent, eutopic endometrium, and PF from individuals with and without endometriosis. However, only 9/16 PF cell samples (endometriosis, n* *=* *5; no endometriosis, n* *=* *4) stained positive for BW495/36, an antibody that discriminates between endometrial epithelium and pelvic mesothelium (van der Linden *et al.*, 1995).

Finally, Masuda *et al.* (2021) investigated for the first time the presence of endometrial mesenchymal stem cells and epithelial progenitors in menstrual blood and PF on cycle Days 2 or 3 and in the non-menstrual phase. By clonogenic assay and flow cytometry, endometrial mesenchymal stem cells were reported during menstruation in 77% of the participants with endometriosis and in 44% of those without. The corresponding figures for epithelial progenitor cells were 60% and 25%, respectively. More clonogenic cells were found in women with endometriosis than in those without the condition also when PF was collected in the non-menstrual phase (Masuda *et al.*, 2021). The trends appear suggestive, although not significant, and would support both the Sampson's and stem cell hypotheses of endometriosis causation.

### Frequency, amount, and composition of RM and differences between primates with and without endometriosis

Retrograde menstruation in animals was described only in primates (D’Hooghe *et al.*, 1991, 1996a). A total of two original studies published in the 1990s were included in this review (Fig. 1B). The studies were conducted in Kenya by a Belgian group (D’Hooghe *et al.*, 1991, 1996b).

In 1991, D’Hooghe *et al.,* evaluating spontaneous endometriosis in 52 baboons, reported the presence of retrograde menstruation in 33% of animals that underwent laparoscopy during menses (D’Hooghe *et al.*, 1991). However, the aspect or composition of the retrograde menstruation was not described. A few years later, the same authors demonstrated that PF was 10 times more frequently blood-stained during menses (62%) than during non-menstrual phases (6%). Retrograde menstruation was observed more frequently in animals with naturally occurring endometriosis (83%) than in animals with a normal pelvis (51%) (D’Hooghe *et al.*, 1996b). Although studies on baboons strongly suggest that baboons can develop some degrees of endometriosis if they are regularly exposed to retrograde menstruation, the characteristics of this phenomenon have not been described extensively in terms of prevalence, composition, and amount (Cornillie *et al.*, 1992; D’Hooghe *et al.*, 1996b).

### Bias and quality analysis

A formal methodological quality assessment for human studies was completed using the NOS. Five studies did not describe the appropriate method of PF assessment. Two of the studies collected menstrual samples at different cycle phases. A breakdown of the NOS scoring is presented in Supplementary Table S1. Given the limited number of studies considered in animals, the NOS was not performed for them.

## Discussion

**Figure 2.**
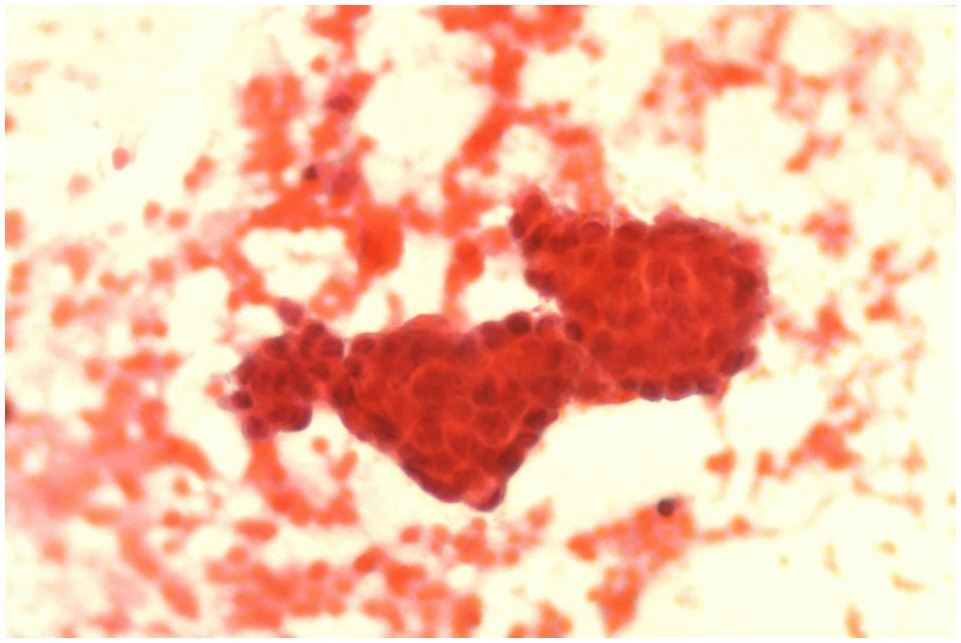
**Photomicrograph showing a gland-like cluster of endometrial cells with lysed erythrocytes in the background**. Peritoneal fluid was collected from the posterior cul-de-sac during a laparoscopy performed in the immediate postmenstrual phase (×100; Papanicolaou stain).

The extreme quantitative and qualitative heterogeneity of the studies considered in this review make it difficult to draw definitive conclusions. Retrograde bleeding is not synonymous with RM, as menstruation implies the presence of endometrial cells and glands in addition to blood (Fig. 2). In fact, the earlier studies in particular relied mainly or entirely on the color of the PF, which presumably grossly reflects the concentration of erythrocytes, as a proxy for RM. Furthermore, the presence of endometriosis could theoretically have acted as a confounding factor, since a higher frequency of endometrial cells in the PF of patients with the disease could be the result not only of an increase in RM but also of shedding from established endometriotic lesions (Kruitwagen, 1993; Sharpe-Timms, 2005). However, endometrial cells have sometimes been found with the same frequency in the PF of menstruators with and without endometriosis (e.g. Koninckx *et al.*, 1980). In any case, the question of the cellular source causing the original ectopic endometrial implantation would remain. On the other hand, paradoxically, the very individuals with the most extensive inflammation-induced mesothelial damage, i.e. those with the highest likelihood of endometrial cell adhesion to the extracellular matrix and the highest risk of endometriosis onset and progression, may have been those with a reduced number of free-floating cells in the PF pool, thus potentially nullifying or even reversing an initially positive relationship between the number of endometrial cells or fragments detected in the PF and the presence of pelvic endometriosis.

For each study participant, laparoscopy allowed assessment of the RM phenomenon on a single occasion and during a specific cycle phase. Therefore, no data are available to describe the course of RM within an entire cycle and between subsequent cycles. Populations with very different risks of endometriosis, such as infertile or parous subjects undergoing tubal ligation, were selected. The indication for laparoscopy was not always clear for all the participants. Individuals under hormonal treatments were not always excluded, and the use of such medications was not systematically reported. Sometimes cases with endometriosis were categorized as yes versus no without stratification of PF findings based on different disease stages. Occasionally, the proportion of patients with endometriosis was not reported. The description of the pelvic findings in controls was sometimes inadequate, and tubal patency was not always confirmed in all subjects.

Samples of PF were often collected at different phases of the cycle and not systematically during menses. This may have influenced the results, especially if refluxed endometrial cells adhere to pelvic structures within a few hours or days and thus are not reliably quantifiable after the menstrual phase. Many studies classified the presence of endometrial cells as yes or no without attempting to quantify cell components. In some studies, PF was collected after tubal flushing or intrauterine maneuvers performed immediately before or a few days prior to laparoscopy.

Importantly, completely different modalities of endometrial cell detection were used, including cytological staining, cell block analysis, immunocytochemistry, cell culture alone versus cell culture with colony counting, different methods of cell culture or preparation of a cell monolayer, sometimes without definitive demonstration of the endometrial origin of the cells observed in culture. Indeed, according to Bokor *et al.* (2009), ‘it is very difficult to identify with 100% certainty specific PF cell types by specific immunocytological markers, since endometrial epithelial, endometrial stromal, mesothelial cells, and macrophages all stain positively for more than one marker’ (Bokor *et al.*, 2009).

Regarding biometric considerations, most studies had limited or very limited sample sizes. Therefore, inferences were sometimes drawn based on the lack of statistical significance of the adopted tests, when the absence of differences could have been due to type II errors. In fact, a preplanned power calculation was almost never described. The most striking finding that casts a shadow of doubt over the body of evidence on RM is the extreme inconsistency of prevalence estimates of PF endometrial cells detected by cytology, immunocytochemistry, or cell culture among different studies.

Finally, the between-study heterogeneity described above prevented a quantitative synthesis of the results, which theoretically could have somewhat overcome the problem of the limited sample size of most of the studies.

### Why do only 3–5% of menstruators develop endometriosis? Which pathogenic model is biologically plausible?

Most researchers believe that endometriosis originates from the endometrium via RM. In this vein, several investigators have recently begun to bring under the spotlight the impressive increase, over the past two centuries, in the number of ovulatory menstruations that occur in the period between the progressively anticipated menarche and the delayed first full-term pregnancy (Jarrell *et al.*, 2016; Jarrell, 2018; Fathalla, 2019; Scioscia *et al.*, 2019; Yovich *et al.*, 2020; Pei *et al.*, 2022; Guo, 2023; Mumusoglu and Hsueh, 2023; Vercellini *et al.*, 2023). Thus, it is probably time to focus also on this macroscopic epidemiologic change, in addition to scrutinizing the numerous potential molecular and cellular pathogenic mechanisms that have been proposed in recent decades but have not yet led to a convincing and consistent clarification of the actual cause(s) of endometriosis.

However, even among the proponents of this evolutionary view, almost all experts believe that RM is only the ‘*primum movens*’ in the sequence of events leading to endometriosis, but that other causative factors would be necessary for full-blown disease forms to manifest. In other words, RM would merely transport endometrial cells or fragments into the pelvis, thus providing the biological substrate upon which the true causative factor(s) would act, activating those biomolecular and immunological processes that would allow dislocated endometrial cells or fragments, otherwise destined for necrosis and resorption, not only to adhere to the extracellular matrix, but also to escape macrophage digestion (Kuan *et al.*, 2021), induce angiogenesis, avoid or delay apoptosis and trigger inflammation and fibrosis (Parazzini *et al.*, 2017; Lagana *et al.*, 2019).

This belief is based on the hypothesis that RM is a universal, physiological phenomenon that occurs similarly in almost all menstruators during the reproductive period, and on the postulate that if RM were the sole etiology of endometriosis, then all menstruators would develop endometriosis. Deductively, additional causative factors, ‘that are not biologically related to RM *per se*, but acting on refluxed endometrial cells/fragments’, are indispensable for the development of the disease (Parazzini *et al.*, 2017; Lagana *et al.*, 2019). Only exposure to these factors would explain why only about 1 in 20 women with regular RM develops overt endometriosis (Ghiasi *et al.*, 2020; Parazzini *et al.*, 2020; Sarria-Santamera *et al.*, 2020).

Within this multifactorial, multistage, stepwise deductive construct, the concept, very schematically synthesized, that between the two components of RM, the true pelvic aggressor is the endometrial cell, independent of the erythrocytes, holds a crucial position, because only those aberrant endometrial cells that are either inherently more aggressive in terms of adhesion, angiogenesis, infiltration, and mitotic potential, or that are secondarily rendered so by additional causative factors, would be able to implant and thrive at ectopic sites (Ulukus *et al.*, 2006). This long-standing view also includes the so-called ‘endometrial determinism’ hypothesis (Viganò *et al.*, 2023). However, according to a recent comprehensive critical assessment of the available experimental evidence, endometrial abnormalities found in individuals with endometriosis appear to be a consequence, rather than a cause, of endometriosis and may well be induced secondarily by the disease itself (Guo *et al.*, 2023).

Indirect proof supporting the theory of endometrial determinism is the occurrence of ‘natural autotransplant experiments’ in individuals without classic endometriotic lesions, that is, in individuals without a predisposition for eutopic endometrium to implant in the pelvis (Marras *et al.*, 2019). One such example is the frequently observed growth of endometriotic nodules within the abdominal wall after a previous cesarean delivery that implied the dissemination of decidual fragments between the rectus muscles and the fascia or in the subcutaneous fat layer (Horton *et al.*, 2008). The same is true for most cases of post-cesarean bladder endometriosis (Vercellini *et al.*, 2002).

However, the available inconsistent data do not allow a definitive conclusion that RM is a universal phenomenon occurring similarly in almost all menstruators, and a simpler reductionist pathogenic model should be rejected first. The basic concept here would be that RM may not occur with similar quantitative, qualitative, and temporal patterns in almost all menstruators, and that differences in these characteristics may *per se* determine the degree of risk of adhesion, survival, and proliferation endometrial cells/fragments at ectopic sites, and thus promotion of full-blown disease forms (Nap *et al.*, 2003; Vercellini *et al.*, 2024a,b). Indeed, the amount of hemoperitoneum observed in patients with acute pelvic pain was found to be associated with the likelihood of subsequent development of infiltrating fibrotic endometriotic lesions (Bean *et al.*, 2019).

Within this alternative reductionist model, features of pelvic iron metabolism that facilitate adhesion and progression toward infiltration and fibrosis should be considered critical (Nap *et al.*, 2003). The concept that pelvic hemoglobin overload may lead to multiple cytotoxic effects due to the pro-oxidant and pro-inflammatory potential of heme, its non-protein moiety with ferrous iron core, was originally developed by the group of Jacques Donnez and subsequently confirmed by several independent researchers and systematic literature reviews (Van Langendonckt *et al.*, 2002a,b; Defrère *et al.*, 2008; Bokor *et al.*, 2009; Lousse *et al.*, 2009, 2012; Pirdel and Pirdel, 2014; Donnez *et al.*, 2016; Ng *et al.*, 2020; Ansariniya *et al.*, 2022; Wyatt *et al.*, 2023). Moreover, heme can induce local progesterone resistance (Ma *et al.*, 2023).

When the amount of refluxed erythrocytes exceeds the phagocytic and clearance capacity of PF macrophages, firstly, ferrous iron-induced reactive oxygen species are generated and the delicate mesothelial cell layer may be damaged, with exposure of the extracellular matrix. This would pave the way for adhesion of displaced, viable endometrial cells or fragments, that would not otherwise occur (Koks *et al.*, 2000; Dunselman *et al.*, 2001). The greater the number of erythrocytes, the greater the likelihood of loss of mesothelial integrity, adhesion of normal endometrial cells, and risk of endometriosis *initiation*. Secondly, repeated RM regularly for long periods of time uninterrupted by multiple pregnancies and prolonged exclusive breastfeeding (i.e. the evolutionary factor), would accelerate disease *promotion*, based on the local metabolic and endocrine activities of ectopic endometrial implants (Bulun *et al.*, 2019). Thirdly, prolonged exposure to excessive ferrous iron and interactions with ferroptosis pathways would exacerbate inflammation, induce fibrosis, and facilitate survival and infiltration of ectopic endometrial cells, thereby fostering disease *progression* (Ng *et al.*, 2020; Li *et al.*, 2021, 2022, 2023; Zhang *et al.*, 2022; Huang *et al.*, 2023; Kobayashi *et al.*, 2023; Liu *et al.*, 2023; Wyatt *et al.*, 2023).

In this reductionist model, the real pelvic aggressor would be the red blood cell, rather than the endometrial cell, and external biological determinants (e.g. genetic predisposition, epigenetic modulation, somatic mutations, exposure to endocrine-disrupting chemicals or to infectious agents, and nutrition) would not be indispensable. They could still play a role (Parazzini *et al.*, 2017; Shafrir *et al.*, 2018; Lagana *et al.*, 2019), but probably as optional aggravating variables rather than primary causative factors. For example, modifiable factors such as smoking, diet, exercise, stress, weight fluctuations and exposure to endocrine-disrupting chemicals, and non-modifiable factors such as age, ethnicity, individual genetic background, and hyper-oestrogenic and/or hypo-oestrogenic intrauterine exposure, may indirectly affect the risk of endometriosis by influencing age at menarche and the menstrual pattern during the following years, and thus the overall RM amount during the early reproductive period (Kuan *et al.*, 2021; Liang *et al.*, 2023; Vercellini *et al.*, 2023). Moreover, the occurrence of somatic KRAS mutation in basal cells of eutopic endometrial glands (Inoue *et al.*, 2019; Bulun, 2022; Praetorius *et al.*, 2022; Bulun *et al.*, 2023) may modulate the ferroptosis pathway, with modalities to be further elucidated (Bartolacci *et al.*, 2022; Müller *et al.*, 2023), hypothetically explaining the greater anatomic disease severity observed in patients with KRAS mutations in endometriotic lesions (Orr *et al.*, 2023).

### Is the notion of retrograde menstruation as a universal physiological phenomenon an endometriosis dogma?

Based on the results of the present review, one might wonder whether an acritically accepted academic notion was born after the publication of the seminal papers by Blumenkrantz *et al.* (1981) and Halme *et al.* (1984; a total of 841 citations according to Scopus; accessed on 21 November 2023) (Blumenkrantz *et al.*, 1981; Halme *et al.*, 1984). The repetition of a statement increases the perception of its factuality (Hasher *et al.*, 1977). The ‘illusory truth effect’ or ‘reiteration effect’ has also been attributed to the increased credibility of information that appears familiar (Begg *et al.*, 1992).

The extreme variability in the reported percentages of PF samples positive for endometrial cells or fragments should in itself raise fundamental doubts about where the truth lies. Furthermore, as with estimates for endometriosis prevalence rates, we mostly have information on specific subgroups of individuals, such as patients with infertility or pelvic pain, which may not be representative of the general population.

Thus, two pathogenic models can be contrasted. On the one hand, a multifactorial, multistage, stepwise model in which the pelvic aggressor is the endometrial cell independent of the total amount of RM. Only those women with particularly ‘aggressive’ endometrium capable of infiltrating the extracellular matrix without the need for prior damage to the mesothelium would develop endometriosis. On the other hand, according to the reductionist model, the pelvic aggressor is the erythrocytes, which would allow oxidative breakdown of the mesothelium as a precondition for the adhesion and implantation of normal endometrial cells. In this model, only those individuals with the largest amounts of refluxed blood would develop endometriosis, regardless of the presence of aberrations in refluxed endometrial cells.

In their excellent critical analysis of the evidence, D’Hooghe and Debrock (2002) concluded, ‘It is not proven that retrograde menstruation is a universal phenomenon occurring similarly in women with and without endometriosis’. Thus, it cannot be excluded that the overall iron-related pelvic oxidative stress (Bulletti *et al.*, 2002; Van Langendonckt *et al.*, 2002a,b; Defrère *et al.*, 2008; Bokor *et al.*, 2009; Lousse *et al.*, 2009, 2012; Pirdel and Pirdel, 2014; Donnez *et al.*, 2016; Bulun *et al.*, 2019; Ng *et al.*, 2020; Ansariniya *et al.*, 2022; Wyatt *et al.*, 2023), is sufficient in itself to explain the development of endometriosis. In addition, the reductionist model accounts for the impressive increase in the number of ovulatory menses observed in the post-industrial era, which, according to several investigators, should not be considered physiological (Jarrell *et al.*, 2016; Jarrell, 2018; Fathalla, 2019; Scioscia *et al.*, 2019; Yovich *et al.*, 2020; Pei *et al.*, 2022; Guo, 2023; Mumusoglu and Hsueh, 2023; Vercellini *et al.*, 2023).

In conclusion, due to several methodological limitations of the scarce available evidence, none of the issues that prompted the present review can be reliably disentangled, and we can neither accept nor reject the original Sampson model of RM as a necessary and sufficient cause for the development of endometriosis. After a critical evaluation of the published data, one single fact seems to stand out: the claim that almost all women experience regular and similar RM during the reproductive period may be hypothetically true, but is currently unsubstantiated.

## Supplementary Material

hoae045_Supplementary_Data

## Data Availability

The data included in this article were extracted as published in the available original articles. No new data were generated or analyzed to support this paper.

## References

[hoae045-B1] Allaire C , Bedaiwy MA, Yong PJ. Diagnosis and management of endometriosis. CMAJ 2023;195:E363–E371. 36918177 10.1503/cmaj.220637 PMC10120420

[hoae045-B2] Ansariniya H , Yavari A, Javaheri A, Zare F. Oxidative stress-related effects on various aspects of endometriosis. Am J Reprod Immunol 2022;88:e13593. 35781369 10.1111/aji.13593

[hoae045-B3] Badawy SZ , Cuenca V, Marshall L, Munchback R, Rinas AC, Coble DA. Cellular components in peritoneal fluid in infertile patients with and without endometriosis. Fertil Steril 1984;42:704–708. 6208058

[hoae045-B4] Bartolacci C , Andreani C, Vale G, Berto S, Melegari M, Crouch AC, Baluya DL, Kemble G, Hodges K, Starrett J et al Targeting de novo lipogenesis and the Lands cycle induces ferroptosis in KRAS-mutant lung cancer. Nat Commun 2022;13:4327. 35882862 10.1038/s41467-022-31963-4 PMC9325712

[hoae045-B5] Bartosik D , Jacobs SL, Kelly LJ. Endometrial tissue in peritoneal fluid. Fertil Steril 1986;46:796–800. 3780999 10.1016/s0015-0282(16)49813-4

[hoae045-B6] Bean E , Cutner A, Saridogan E, Wong M, Naftalin J, Jurkovic D. Hemoperitoneum as a precursor of deep pelvic endometriosis: prospective cohort study. Ultrasound Obstet Gynecol 2019;54:389–394. 30677178 10.1002/uog.20222

[hoae045-B7] Begg IM , Anas A, Farinacci S. Dissociation of processes in belief: source recollection, statement familiarity, and the illusion of truth. J Exp Psychol Gen 1992;121:446–458.

[hoae045-B8] Blumenkrantz MJ , Gallagher N, Bashore RA, Tenckhoff H. Retrograde menstruation in women undergoing chronic peritoneal dialysis. Obstet Gynecol 1981;57:667–670. 7219918

[hoae045-B9] Bokor A , Debrock S, Drijkoningen M, Goossens W, Fülöp V, D’Hooghe T. Quantity and quality of retrograde menstruation: a case control study. Reprod Biol Endocrinol 2009;7:123. 19878540 10.1186/1477-7827-7-123 PMC2789082

[hoae045-B10] Bulletti C , De Ziegler D, Polli V, Del Ferro E, Palini S, Flamigni C. Characteristics of uterine contractility during menses in women with mild to moderate endometriosis. Fertil Steril 2002;77:1156–1161. 12057721 10.1016/s0015-0282(02)03087-x

[hoae045-B11] Bulun SE. Endometriosis caused by retrograde menstruation: now demonstrated by DNA evidence. Fertil Steril 2022;118:535–536. 36116802 10.1016/j.fertnstert.2022.07.012

[hoae045-B12] Bulun SE , Yildiz S, Adli M, Chakravarti D, Parker JB, Milad M, Yang L, Chaudhari A, Tsai S, Wei JJ et al Endometriosis and adenomyosis: shared pathophysiology. Fertil Steril 2023;119:746–750. 36925057 10.1016/j.fertnstert.2023.03.006

[hoae045-B13] Bulun SE , Yilmaz BD, Sison C, Miyazaki K, Bernardi L, Liu S, Kohlmeier A, Yin P, Milad M, Wei J. Endometriosis. Endocr Rev 2019;40:1048–1079. 30994890 10.1210/er.2018-00242 PMC6693056

[hoae045-B14] Cornillie FJ , D’Hooghe TM, Bambra CS, Lauweryns JM, Isahakia M, Koninckx PR. Morphological characteristics of spontaneous endometriosis in the baboon (*Papio anubis* and *Papio cynocephalus*). Gynecol Obstet Invest 1992;34:225–228. 1487181 10.1159/000292766

[hoae045-B15] Davis RH , McDonald J, Kyriazis GA, Schneider HP. The peritoneal fluid cytology of the adult female Rhesus monkey. Experientia 1973;29:1242. 4202362 10.1007/BF01935096

[hoae045-B16] Defrère S , Lousse JC, González-Ramos R, Colette S, Donnez J, Van Langendonckt A. Potential involvement of iron in the pathogenesis of peritoneal endometriosis. Mol Hum Reprod 2008;14:377–385. 18508952 10.1093/molehr/gan033

[hoae045-B92] D’Hooghe TM, Bambra CS, Cornillie FJ, Isahakia M, Koninckx PR. Prevalence and laparoscopic appearance of spontaneous endometriosis in the baboon (Papio anubis, Papio cynocephalus). Biol Reprod 1991;45:411–416. 1838282 10.1095/biolreprod45.3.411

[hoae045-B17] D’Hooghe TM , Bambra CS, De Jonge I, Lauweryns JM, Koninckx PR. The prevalence of spontaneous endometriosis in the baboon (*Papio anubis*, *Papio cynocephalus*) increases with the duration of captivity. Acta Obstet Gynecol Scand1996a;75:98–101. 8604618 10.3109/00016349609033298

[hoae045-B18] D’Hooghe TM , Bambra CS, Raeymaekers BM, De Jonge I, Lauweryns JM, Koninckx PR. Intrapelvic injection of menstrual endometrium causes endometriosis in baboons (*Papio cynocephalus* and *Papio anubis*). Am J Obstet Gynecol 1995;173:125–134. 7631669 10.1016/0002-9378(95)90180-9

[hoae045-B19] D’Hooghe TM , Bambra CS, Raeymaekers BM, Koninckx PR. Increased prevalence and recurrence of retrograde menstruation in baboons with spontaneous endometriosis. Hum Reprod1996b;11:2022–2025. 8921084 10.1093/oxfordjournals.humrep.a019537

[hoae045-B20] D’Hooghe TM , Bambra CS, Suleman MA, Dunselman GA, Evers HL, Koninckx PR. Development of a model of retrograde menstruation in baboons (*Papio anubis*). Fertil Steril 1994;62:635–638. 8062962

[hoae045-B21] D’Hooghe TM , Bambra CS, Xiao L, Peixe K, Hill JA. Effect of menstruation and intrapelvic injection of endometrium on inflammatory parameters of peritoneal fluid in the baboon (*Papio anubis* and *Papio cynocephalus*). Am J Obstet Gynecol 2001;184:917–925. 11303199 10.1067/mob.2001.111715

[hoae045-B22] D’Hooghe TM , Debrock S. Endometriosis, retrograde menstruation and peritoneal inflammation in women and in baboons. Hum Reprod Update 2002;8:84–88. 11866244 10.1093/humupd/8.1.84

[hoae045-B23] Donnez J , Binda MM, Donnez O, Dolmans MM. Oxidative stress in the pelvic cavity and its role in the pathogenesis of endometriosis. Fertil Steril 2016;106:1011–1017. 27521769 10.1016/j.fertnstert.2016.07.1075

[hoae045-B24] Dorien FO , Roskams T, Van den Eynde K, Vanhie A, Peterse DP, Meuleman C, Tomassetti C, Peeraer K, D’Hooghe TM, Fassbender A. The presence of endometrial cells in peritoneal fluid of women with and without endometriosis. Reprod Sci 2017;24:242–251. 27324432 10.1177/1933719116653677

[hoae045-B25] Dunselman GA , Groothuis PG, de Goeij AF, Evers JL. The mesothelium, teflon or velcro? Mesothelium in endometriosis pathogenesis. Hum Reprod 2001;16:605–607. 11278202 10.1093/humrep/16.4.605

[hoae045-B26] Fathalla MF. Impact of reproductive evolutionary mismatch on women’s health and the need for action and research. Int J Gynaecol Obstet 2019;144:129–134. 30341890 10.1002/ijgo.12694

[hoae045-B27] Filby CE , Rombauts L, Montgomery GW, Giudice LC, Gargett CE. Cellular origins of endometriosis: towards novel diagnostics and therapeutics. Semin Reprod Med 2020;38:201–215. 33176364 10.1055/s-0040-1713429

[hoae045-B28] Ghiasi M , Kulkarni MT, Missmer SA. Is endometriosis more common and more severe than it was 30 years ago? J Minim Invasive Gynecol 2020;27:452–461. 31816389 10.1016/j.jmig.2019.11.018

[hoae045-B29] Guo SW. How do women get endometriosis? Reprod Biomed Online 2023;48:103696. 38123408 10.1016/j.rbmo.2023.103696

[hoae045-B30] Guo SW , Habiba M, Benagiano G. From retrograde menstruation to endometrial determinism and a brave new world of “Root Treatment” of endometriosis: destiny or a fanciful utopia? Biomolecules 2023;13:336. 36830705 10.3390/biom13020336 PMC9953699

[hoae045-B31] Halme J , Hammond MG, Hulka JF, Raj SG, Talbert LM. Retrograde menstruation in healthy women and in patients with endometriosis. Obstet Gynecol 1984;64:151–154. 6234483

[hoae045-B32] Hasher L , Goldstein D, Toppino T. Frequency and the conference of referential validity. J Verbal Learn Verbal Behav 1977;16:107–112.

[hoae045-B33] Horton JD , Dezee KJ, Ahnfeldt EP, Wagner M. Abdominal wall endometriosis: a surgeon’s perspective and review of 445 cases. Am J Surg 2008;196:207–212. 18513698 10.1016/j.amjsurg.2007.07.035

[hoae045-B34] Huang X , Song Y, Wei L, Guo J, Xu W, Li M. The emerging roles of ferroptosis in organ fibrosis and its potential therapeutic effect. Int Immunopharmacol 2023;116:109812. 36746022 10.1016/j.intimp.2023.109812

[hoae045-B35] Inoue S , Hirota Y, Ueno T, Fukui Y, Yoshida E, Hayashi T, Kojima S, Takeyama R, Hashimoto T, Kiyono T et al Uterine adenomyosis is an oligoclonal disorder associated with KRAS mutations. Nat Commun 2019;10:5785. 31857578 10.1038/s41467-019-13708-y PMC6923389

[hoae045-B36] Kobayashi H , Yoshimoto C, Matsubara S, Shigetomi H, Imanaka S. Current understanding of and future directions for endometriosis-related infertility research with a focus on ferroptosis. Diagnostics 2023;13:1926. 37296777 10.3390/diagnostics13111926 PMC10252275

[hoae045-B37] Koks CA , Demir Weusten AY, Groothuis PG, Dunselman GA, de Goeij AF, Evers JL. Menstruum induces changes in mesothelial cell morphology. Gynecol Obstet Invest 2000;50:13–18. 10895021 10.1159/000010271

[hoae045-B38] Koninckx PR , Ide P, Vandenbroucke W, Brosens IA. New aspects of the pathophysiology of endometriosis and associated infertility. J Reprod Med 1980;24:257–260. 7420327

[hoae045-B39] Kruitwagen RF. Menstruation as the pelvic aggressor. Baillieres Clin Obstet Gynaecol 1993;7:687–700. 8131310 10.1016/s0950-3552(05)80458-4

[hoae045-B40] Kruitwagen RF , Poels LG, Willemsen WN, de Ronde IJ, Jap PH, Rolland R. Endometrial epithelial cells in peritoneal fluid during the early follicular phase. Fertil Steril 1991;55:297–303. 1991528 10.1016/s0015-0282(16)54119-3

[hoae045-B41] Kuan KKW , Gibson DA, Whitaker LHR, Horne AW. Menstruation dysregulation and endometriosis development. Front Reprod Health 2021;3:756704. 36304032 10.3389/frph.2021.756704 PMC9580640

[hoae045-B42] Kulenthran A , Jeyalakshmi N. Dissemination of endometrial cells at laparoscopy and chromotubation—a preliminary report. Int J Fertil 1989;34:256–258. 2570762

[hoae045-B43] Jarrell J. The significance and evolution of menstruation. Best Pract Res Clin Obstet Gynaecol 2018;50:18–26. 29530426 10.1016/j.bpobgyn.2018.01.007

[hoae045-B44] Jarrell J , Arendt-Nielsen L. Evolutionary considerations in the development of chronic pelvic pain. Am J Obstet Gynecol 2016;215:201.e1–201.e4. 10.1016/j.ajog.2016.05.019 27269450

[hoae045-B45] Lagana AS , Garzon S, Gotte M, Viganò P, Franchi M, Ghezzi F, Martin DC. The pathogenesis of endometriosis: molecular and cell biology insights. Int J Mol Sci 2019;20:5615. 31717614 10.3390/ijms20225615 PMC6888544

[hoae045-B46] Li B , Duan H, Wang S, Li Y. Ferroptosis resistance mechanisms in endometriosis for diagnostic model establishment. Reprod Biomed Online 2021;43:127–138. 33992553 10.1016/j.rbmo.2021.04.002

[hoae045-B47] Li G , Lin Y, Zhang Y, Gu N, Yang B, Shan S, Liu N, Ouyang J, Yang Y, Sun F et al Endometrial stromal cell ferroptosis promotes angiogenesis in endometriosis. Cell Death Discov 2022;8:29. 35039492 10.1038/s41420-022-00821-z PMC8763888

[hoae045-B48] Li Y , He Y, Cheng W, Zhou Z, Ni Z, Yu C. Double-edged roles of ferroptosis in endometriosis and endometriosis-related infertility. Cell Death Discov 2023;9:306. 37607902 10.1038/s41420-023-01606-8 PMC10444804

[hoae045-B49] Liang J , Ali F, Ramalyer M, Borahay MA. Determinants of menstrual blood flow. Curr Epidemiol Rep 2023;10:210–220. 38275001 10.1007/s40471-023-00332-0 PMC10810143

[hoae045-B50] Liu DT , Hitchcock A. Endometriosis: its association with retrograde menstruation, dysmenorrhoea and tubal pathology. Br J Obstet Gynaecol 1986;93:859–862. 3741813 10.1111/j.1471-0528.1986.tb07995.x

[hoae045-B51] Liu M , Wu K, Wu Y. The emerging role of ferroptosis in female reproductive disorders. Biomed Pharmacother 2023;166:115415. 37660655 10.1016/j.biopha.2023.115415

[hoae045-B52] Lousse JC , Defrère S, Van Langendonckt A, Gras J, González-Ramos R, Colette S, Donnez J. Iron storage is significantly increased in peritoneal macrophages of endometriosis patients and correlates with iron overload in peritoneal fluid. Fertil Steril 2009;91:1668–1675. 18396284 10.1016/j.fertnstert.2008.02.103

[hoae045-B53] Lousse JC , Van Langendonckt A, Defrere S, Ramos RG, Colette S, Donnez J. Peritoneal endometriosis is an inflammatory disease. Front Biosci (Elite Ed) 2012;4:23–40. 22201853 10.2741/e358

[hoae045-B54] Ma XQ , Liu YY, Zhong ZQ, Chen SM, Hu WT, Sheng YR, Liu YK, Wei CY, Li MQ, Zhu XY. Heme induced progesterone-resistant profiling and promotion of endometriosis in vitro and in vivo. Biochim Biophys Acta Mol Basis Dis 2023;1869:166761. 37247698 10.1016/j.bbadis.2023.166761

[hoae045-B55] Marras S , Pluchino N, Petignat P, Wenger JM, Ris F, Buchs NC, Dubuisson J. Abdominal wall endometriosis: an 11-year retrospective observational cohort study. Eur J Obstet Gynecol Reprod Biol X 2019;4:100096. 31650130 10.1016/j.eurox.2019.100096 PMC6804734

[hoae045-B56] Masuda H , Schwab KE, Filby CE, Tan CSC, Tsaltas J, Weston GC, Gargett CE. Endometrial stem/progenitor cells in menstrual blood and peritoneal fluid of women with and without endometriosis. Reprod Biomed Online 2021;43:3–13. 34011465 10.1016/j.rbmo.2021.04.008

[hoae045-B57] Müller F , Lim JKM, Bebber CM, Seidel E, Tishina S, Dahlhaus A, Stroh J, Beck J, Yapici FI, Nakayama K et al Elevated FSP1 protects KRAS-mutated cells from ferroptosis during tumor initiation. Cell Death Differ 2023;30:442–456. 36443441 10.1038/s41418-022-01096-8 PMC9950476

[hoae045-B58] Mumusoglu S , Hsueh AJW. Is endometriosis due to evolutionary maladaptation? Reprod Biomed Online 2023;48:103695. 10.1016/j.rbmo.2023.103695. 38177037

[hoae045-B59] Nap AW , Groothuis PG, Demir AY, Maas JW, Dunselman GA, de Goeij AF, Evers JL. Tissue integrity is essential for ectopic implantation of human endometrium in the chicken chorioallantoic membrane. Hum Reprod 2003;18:30–34. 12525437 10.1093/humrep/deg033

[hoae045-B60] Ng SW , Norwitz SG, Taylor HS, Norwitz ER. Endometriosis: the role of iron overload and ferroptosis. Reprod Sci 2020;27:1383–1390. 32077077 10.1007/s43032-020-00164-z

[hoae045-B61] Novak E. The significance of uterine mucosa in the fallopian tube, with a discussion of the origin of aberrant endometrium. Am J Obstet Gynecol 1926;12:484–526.

[hoae045-B62] Novak E. Pelvic endometriosis and its treatment. Am J Surg 1936;33:422–421.

[hoae045-B63] Orr NL , Albert A, Liu YD, Lum A, Hong J, Ionescu CL, Senz J, Nazeran TM, Lee AF, Noga H et al KRAS mutations and endometriosis burden of disease. J Pathol Clin Res 2023;9:302–312. 36977195 10.1002/cjp2.317 PMC10240146

[hoae045-B64] Parazzini F , Esposito G, Tozzi L, Noli S, Bianchi S. Epidemiology of endometriosis and its comorbidities. Eur J Obstet Gynecol Reprod Biol 2017;209:3–7. 27216973 10.1016/j.ejogrb.2016.04.021

[hoae045-B65] Parazzini F , Roncella E, Cipriani S, Trojano G, Barbera V, Herranz B, Colli E. The frequency of endometriosis in the general and selected populations: a systematic review. J Endometr Pelv Pain Disord 2020;12:176–189.

[hoae045-B66] Pei Z , Lu W, Feng Y, Xu C, Hsueh AJW. Out of step societal and Darwinian adaptation during evolution is the cause of multiple women’s health issues. Hum Reprod 2022;37:1959–1969. 35881063 10.1093/humrep/deac156

[hoae045-B67] Pirdel L , Pirdel M. Role of iron overload-induced macrophage apoptosis in the pathogenesis of peritoneal endometriosis. Reproduction 2014;147:R199–R207. 24599836 10.1530/REP-13-0552

[hoae045-B68] Praetorius TH , Leonova A, Lac V, Senz J, Tessier-Cloutier B, Nazeran TM, Köbel M, Grube M, Kraemer B, Yong PJ et al Molecular analysis suggests oligoclonality and metastasis of endometriosis lesions across anatomically defined subtypes. Fertil Steril 2022;118:524–534. 35715244 10.1016/j.fertnstert.2022.05.030

[hoae045-B69] Reti LL , Byrne GD, Davoren RA. The acute clinical features of retrograde menstruation. Aust N Z J Obstet Gynaecol 1983;23:51–52. 6223617 10.1111/j.1479-828x.1983.tb00160.x

[hoae045-B70] Sampson JA. Peritoneal endometriosis due to menstrual dissemination of endometrial tissue into the pelvic cavity. Am J Obstet Gynecol 1927;14:422–469.

[hoae045-B71] Sampson JA. The development of the implantation theory for the origin of peritoneal endometriosis. Am J Obstet Gynecol 1940;40:549–557.

[hoae045-B72] Sarria-Santamera A , Orazumbekova B, Terzic M, Issanov A, Chaowen C, Asúnsolo-Del-Barco A. Systematic review and meta-analysis of incidence and prevalence of endometriosis. Healthcare 2020;9:29. 33396813 10.3390/healthcare9010029 PMC7824417

[hoae045-B73] Scioscia M , Roman H, Somigliana E, Robillard PY. Increasing number of menstruations in recent generations may contribute to the development of endometriosis: an evolutionary view from a critical analysis of National Health data. Hum Reprod 2019;34:2549–2550. 31820787 10.1093/humrep/dez192

[hoae045-B74] Shafrir AL , Farland LV, Shah DK, Harris HR, Kvaskoff M, Zondervan K, Missmer SA. Risk for and consequences of endometriosis: a critical epidemiologic review. Best Pract Res Clin Obstet Gynaecol 2018;51:1–15. 30017581 10.1016/j.bpobgyn.2018.06.001

[hoae045-B75] Sharpe-Timms KL. Haptoglobin expression by shed endometrial tissue fragments found in peritoneal fluid. Fertil Steril 2005;84:22–30. 16009149 10.1016/j.fertnstert.2005.02.014

[hoae045-B76] Tang J , Wang Z, Lu D, Xie Y, Zhang D. Value of early laparoscopic exploration for primary infertile patients with patent fallopian tubes complicated with pelvic effusion. Med Sci Monit 2022;28:e938637. 36518029 10.12659/MSM.938637 PMC9764669

[hoae045-B77] Ulukus M , Cakmak H, Arici A. The role of endometrium in endometriosis. J Soc Gynecol Investig 2006;13:467–476. 10.1016/j.jsgi.2006.07.005 16990031

[hoae045-B78] van der Linden PJ , de Goeij AF, Dunselman GA, van der Linden EP, Ramaekers FC, Evers JL. Expression of integrins and E-cadherin in cells from menstrual effluent, endometrium, peritoneal fluid, peritoneum, and endometriosis. Fertil Steril 1994;61:85–90. 8293849 10.1016/s0015-0282(16)56457-7

[hoae045-B79] van der Linden PJ , Dunselman GA, de Goeij AF, van der Linden EP, Evers JL, Ramaekers FC. Epithelial cells in peritoneal fluid—of endometrial origin? Am J Obstet Gynecol 1995;173:566–570. 7544070 10.1016/0002-9378(95)90283-x

[hoae045-B80] van der Weiden RM , Arentz PW, Veselic M. Endometrial cells in the peritoneal cavity after laparoscopy and chromotubation. J R Soc Med 1992;85:397–398. 1629848 PMC1293546

[hoae045-B81] Van Langendonckt A , Casanas-Roux F, Dolmans MM, Donnez J. Potential involvement of hemoglobin and heme in the pathogenesis of peritoneal endometriosis. Fertil Steril2002a;77:561–570. 11872213 10.1016/s0015-0282(01)03211-3

[hoae045-B82] Van Langendonckt A , Casanas-Roux F, Donnez J. Iron overload in the peritoneal cavity of women with pelvic endometriosis. Fertil Steril2002b;78:712–718. 12372445 10.1016/s0015-0282(02)03346-0

[hoae045-B83] Vercellini P , Bandini V, Viganò P, Di Stefano G, Merli CEM, Somigliana E. Proposal for targeted, neo-evolutionary-oriented, secondary prevention of early-onset endometriosis and adenomyosis. Part I: pathogenic aspects. Hum Reprod 2023;39:1–17. 10.1093/humrep/dead229 PMC10876119 37951243

[hoae045-B84] Vercellini P , Frontino G, Pisacreta A, De Giorgi O, Cattaneo M, Crosignani PG. The pathogenesis of bladder detrusor endometriosis. Am J Obstet Gynecol 2002;187:538–542. 12237623 10.1067/mob.2002.124286

[hoae045-B86] Vercellini P , Salmeri N, Somigliana E, Piccini M, Caprara F, Viganò P, De Matteis S. Müllerian anomalies and endometriosis as potential explanatory models for the retrograde menstruation/implantation and the embryonic remnants/celomic metaplasia pathogenic theories: a systematic review and meta-analysis. Hum Reprod2024a;39:1460–1470. 10.1093/humrep/deae086 PMC11962717 38733102

[hoae045-B85] Vercellini P , Piccini M, Caprara F, Cetera GE, Viganò P, Somigliana E. Potential anatomical determinants of retrograde menstruation: a comprehensive narrative review. Reprod BioMed Online2024b;104345. 10.1016/j.rbmo.2024.104345 39137508

[hoae045-B87] Viganò P , Casalechi M, Vercellini P, Somigliana E. “Shadow of a Doubt”—the pathogenic role of endometrial defects in endometriosis development and endometriosis-associated infertility: robust demonstration of clinical relevance is still urgently needed. Biomolecules 2023;13:651. 37189399 10.3390/biom13040651 PMC10135529

[hoae045-B88] Willemsen WN , Mungyer G, Smets H, Rolland R, Vemer H, Jap P. Behavior of cultured glandular cells obtained by flushing of the uterine cavity. Fertil Steril 1985;44:92–95. 3891426 10.1016/s0015-0282(16)48683-8

[hoae045-B89] Wyatt J , Fernando SM, Powell SG, Hill CJ, Arshad I, Probert C, Ahmed S, Hapangama DK. The role of iron in the pathogenesis of endometriosis: a systematic review. Hum Reprod Open 2023;2023:hoad033. 37638130 10.1093/hropen/hoad033 PMC10457727

[hoae045-B90] Yovich JL , Rowlands PK, Lingham S, Sillender M, Srinivasan S. Pathogenesis of endometriosis: look no further than John Sampson. Reprod Biomed Online 2020;40:7–11. 31836436 10.1016/j.rbmo.2019.10.007

[hoae045-B91] Zhang Y , Liu X, Deng M, Xu C, Zhang Y, Wu D, Tang F, Yang R, Miao J. Ferroptosis induced by iron overload promotes fibrosis in ovarian endometriosis and is related to subpopulations of endometrial stromal cells. Front Pharmacol 2022;13:930614. 36120348 10.3389/fphar.2022.930614 PMC9478936

