# Supplementary material for: Is retrograde menstruation a universal, recurrent, physiological phenomenon? A systematic review of the evidence in humans and non-human primates
Source: Hum Reprod Open. 2024 Jul 12;2024(3):hoae045. doi: 10.1093/hropen/hoae045 (PMC11272177; doi:10.1093/hropen/hoae045)
Supplement: hoae045_Supplementary_Data [file hoae045_supplementary_data.docx]

Supplementary Table S1: Quality assessment of human studies using the NOS Newcastle–Ottawa scoring system.

| **CRITERION** | | **Ascertainment of endometriosis diagnosis** | | **Representativeness of the endometriosis cases** | **Definition of the controls** | **Same method of ascertainment for cases and controls** | | **Quality of population description** | | **Sampling technique** | **TOTAL SCORE** |
| --- | --- | --- | --- | --- | --- | --- | --- | --- | --- | --- | --- |
| **DECISION**  **RULE      STUDY** | | 1 point: The criteria for diagnosing endometriosis were clearly outlined, included the use of surgery.  0 points: The criteria for diagnosing endometriosis were not included. | | 1 point: All stages of endometriosis were included in the cases (I-IV).  0 points: The study focuses on a single phenotype of endometriosis or not specified. | 1 point: Endometriosis was excluded by pelvic surgery in the controls.  0 points: Controls were included without surgical exclusion of endometriosis by pelvic surgery. | 1 point: Cases and controls recruited from the same population group (e.g. age, menstrual cycle phase, patency of tubes).  0 points: Cases and controls were chosen randomly, or the population is not described | | 1 point: The study gives a clear description of the population and uses an appropriate method of collecting peritoneal fluid.  0 points: The study did not report a clear description of the population or appropriate method of peritoneal fluid assessment. | | 1 Same method of ascertainment for cases and controls.  0 Not the same method of ascertainment for case and controls. |  |
| Koninckx *et al.* 1980 | | 1 | | 0 | 1 | 0 | | 0 | | 1 | 3 |
| Blumenkrantz *et al.* 1981 | | 1 | | 0 | 0 | 1 | | 0 | | 1 | 4 |
| Reti *et al.* 1983 | | 1 | | 0 | 1 | 1 | | 1 | | 1 | 5 |
| Halme *et al.* 1984 | | 1 | | 0 | 1 | 1 | | 0 | | 1 | 4 |
| Badawy *et al.* 1984 | | 1 | | 0 | 1 | 1 | | 1 | | 1 | 5 |
| Willemsen *et al.* 1985 | | 1 | | 0 | 1 | 1 | | 1 | | 1 | 5 |
| Liu and Hitchcock 1986 | | 1 | | 0 | 1 | 1 | | 0 | | 1 | 4 |
| Bartosik *et al.* 1986 | | 1 | | 0 | 1 | 1 | | 1 | | 1 | 5 |
| Kruitwagen 1991 | | 1 | | 0 | 1 | 1 | | 1 | | 1 | 5 |
| van der Linden *et al.* 1995 | | 1 | | 0 | 1 | 1 | | 1 | | 1 | 5 |
| Bulletti *et al.* 2002 | | 1 | | 0 | 1 | 1 | | 1 | | 1 | 5 |
| Sharpe-Timms 2005 | | 1 | | 0 | 1 | 0 | | 0 | | 1 | 3 |
| Bokor *et al.* 2009 | | 1 | | 1 | 1 | 1 | | 1 | | 1 | 6 |
| O *et al.* 2017 | | 1 | | 1 | 1 | 1 | | 1 | | 1 | 6 |
| Masuda *et al.* 2021 | | 1 | | 0 | 1 | 1 | | 1 | | 1 | 5 |
| **CRITERION** | | **DECISION RULE / SCORE** | | | |  | |  |  |  |  |
| Ascertainment of endometriosis diagnosis: | | 1 point: The criteria for the diagnosis of endometriosis were clearly described and included the use of one or more of the following techniques, including transvaginal ultrasound, magnetic resonance imaging, and laparoscopy.  0 points: The diagnosis was based on other unvalidated approaches (e.g., chronic pelvic pain), or the diagnostic methods were not described. | | | | | |  |  |  |  |
| Representativeness of the endometriosis cases | | 1 point: All kinds of endometriosis have been included in the study (I-IV stages OR SUP/DIE/OMA)  0 point: The study included a phenotype specific of endometriosis. | | | | | |  |  |  |  |
| Definition of the controls | | 1 point: Endometriosis were excluded based in surgery report;  0 point: Controls were included without a surgery exclusion of endometriosis. | | | | | |  |  |  |  |
| Same method of ascertainment for cases and controls | | 1 point: Yes, cases and controls recruited from the same population group (e.g. age, presence of pain, infertility; menstrual cycle phase) -  0 point: No, patients chosen randomly or not reported | | | | | |  |  |  |  |
| Quality of population description: | | 1 point: The study reported a clear description of the population (e.g. age, BMI, duration of infertility) with proper measures of dispersion (e.g., mean, standard deviation) and ovarian stimulation-embryo transfer protocol.  0 points: The study did not report a clear description of the population or ovarian stimulation-embryo transfer protocol, incompletely reported descriptive statistics, or did not report measures of dispersion. | | | | | |  |  |  |  |
| Sampling technique: | | 1 point: Sampe sampling technique for cases and controls  0 points: Potential convenience sampling or unspecified sampling technique. | | | | | |  |  |  |  |
| The individual components listed above are summed to generate a total modified Newcastle-Ottawa risk of bias score for each study. Total scores range from 0 to 6. For the total score grouping, studies were judged to be of low risk of bias (≥X points) or high risk of bias (<X points). | | | | | | | |  |  |  |  |
